# Accuracy of Matrix-Assisted Laser Desorption Ionization–Time of Flight Mass Spectrometry for Identification of Mycobacteria: a systematic review and meta-analysis

**DOI:** 10.1038/s41598-018-22642-w

**Published:** 2018-03-07

**Authors:** Yan Cao, Lei Wang, Ping Ma, Wenting Fan, Bing Gu, Shaoqing Ju

**Affiliations:** 1 Center of Laboratory Medicine, Affiliated Hospital of Nantong University, Nantong, 226000 China; 2 Department of Histology and Embryology, Xuzhou Medical University, Xuzhou, 221004 China; 3 Department of Laboratory Medicine, Affiliated Hospital of Xuzhou Medical University, Xuzhou, 221002 China; 4 Medical Technology School, Xuzhou Medical University, Xuzhou, 221004 China

## Abstract

*Mycobacterium* species are a significant cause of morbidity and mortality worldwide. The present study was carried out to systematically evaluate the accuracy of Matrix-assisted laser desorption ionization–time of flight mass spectroscopy (MALDI-TOF MS) for the identification of clinical pathogenic mycobacteria. After a rigid selection process, 19 articles involving 2,593 mycobacteria isolates were included. The pooled result agreed with the reference method identification for 85% of the isolates to genus level, with 71% (95% CI of 69% to 72%) correct to the species level. The MALDI-TOF MS correctly identified 92% of the *M.tuberculosis* isolates (95% CI of 0.87 to 0.96), and 68% of *M. bovis*isolates (95% CI of 27% to 100%) to the species level. *Mycobacterium tuberculosis* complex in solid media with reference strains using augmented database showing more accurate identification. The identifying accuracy rate of bioMérieuxVitek MS was slight higher than Bruker MALDI Biotyper (75% vs 72%). However, opposite results were obtained in identifications of *M. fortuitum*, *M. kansasii*, *M. marinum*, and *M. terrae* with these two systems. In summary, our results demonstrate that application of MALDI-TOF MS in clinical pathogenic mycobacteria identification is less satisfactory to date. Increasing need for improvement is important especially at species level.

## Introduction

Mycobacteria are group of pathogens that can cause a wide spectrum of pulmonary and extra-pulmonary infections^1,2^, which continue to be a major public health concern in developing and industrialized countries. *Mycobacterium tuberculosis* complex (MTC) remains the major causes of morbidity and mortality^3^, while non-tuberculous mycobacteria (NTM), are frequent primary or opportunistic pathogens, causing pulmonary infection and lymphadenitis in children, skin disease and other extra-pulmonary infections in immune-compromised individuals^4,5^. Early species- or complex-level identification is of utmost importance to differentiate tuberculosis-causing mycobacteria, for epidemiological, public health, and therapeutic reasons.

Conventionally, identification of mycobacteria has been based on well-established phenotypic traits and biochemical profiles. Regardless of improved culture methods, it’s still time-consuming and difficult for identification of less common species. Recently, molecular assays, including PCR sequencing, and PCR hybridization, have been shown to support phenotypic identification methods or as an additional test performed directly on clinical specimens to enable rapid identification^6,7^. Although these methods are highly specific and greatly improve the turnaround time to identification; evaluations of molecular assays have generally been shown to be restricted to a limited number of *Mycobacterium* species, show variable sensitivity and labor-intensity^8^. So then sequencing of other genomic regions or the whole genome is necessary for complete genotyping. However, it is technically demanding and relatively expensive but rapidly decreasing in cost. According to the limitations encountered with currently available methods for identification, an alternative strategy may become necessary for clinical laboratories to overcome this hurdle.

Matrix-assisted laser desorption ionization-time of flight mass spectrometry (MALDI-TOF MS) is a new type of soft ionization mass spectrometry. An increasing number of clinical microbiological laboratories consider it as an innovative approach for bacterial identification. Our previous study evaluated the use of MALDI-TOF MS for rapid identification of the clinical streptococci^9^. As regards the identification of mycobacteria, lots of studies have identified matrix-assisted laser desorption ionization–time of flight mass spectrometry (MALDI-TOF MS) as a powerful, rapid, and cost-effective method^10–14^. However, those reports differed in types of isolation medium, extraction protocols and libraries used. Additionally, many studies only included a few strains, and some had inconsistent results. The purpose of this study was to evaluate the robust accuracy of MALDI-TOF MS using different systems and culture medium to identify clinically pathogenic mycobacteria to genus and species level, respectively, by performing a meta-analysis that combines a large number of studies to define the reliability of MALDI-TOF MS for this purpose.

## Results

### Eligible studies

After a comprehensive literature search, 128 items were obtained by searching PubMed and EmBase with defined retrieval strings. After manual search and duplicate removal, a total of 86 articles remained for full-text scanning after title and abstract review. Among the excluded articles, 33 were excluded because they were not pertinent to the present study, including 3 case reports, 11 reviews and 6 posters. After the papers were screened, 2 studies were excluded because organisms were not isolated from humans; 16 were discarded as a result of technological innovation; 9 were excluded because they concerned identification of drug resistance; 21 were rejected because of the lack of reference method or detailed description of isolates; 19 were excluded because they reported mass spectrometry technique other than MALDI-TOF or because the identification of clinical mycobacteria were unrelated. As a result, 19 articles were included in this meta-analysis (Fig. 1).

**Figrue 1:**
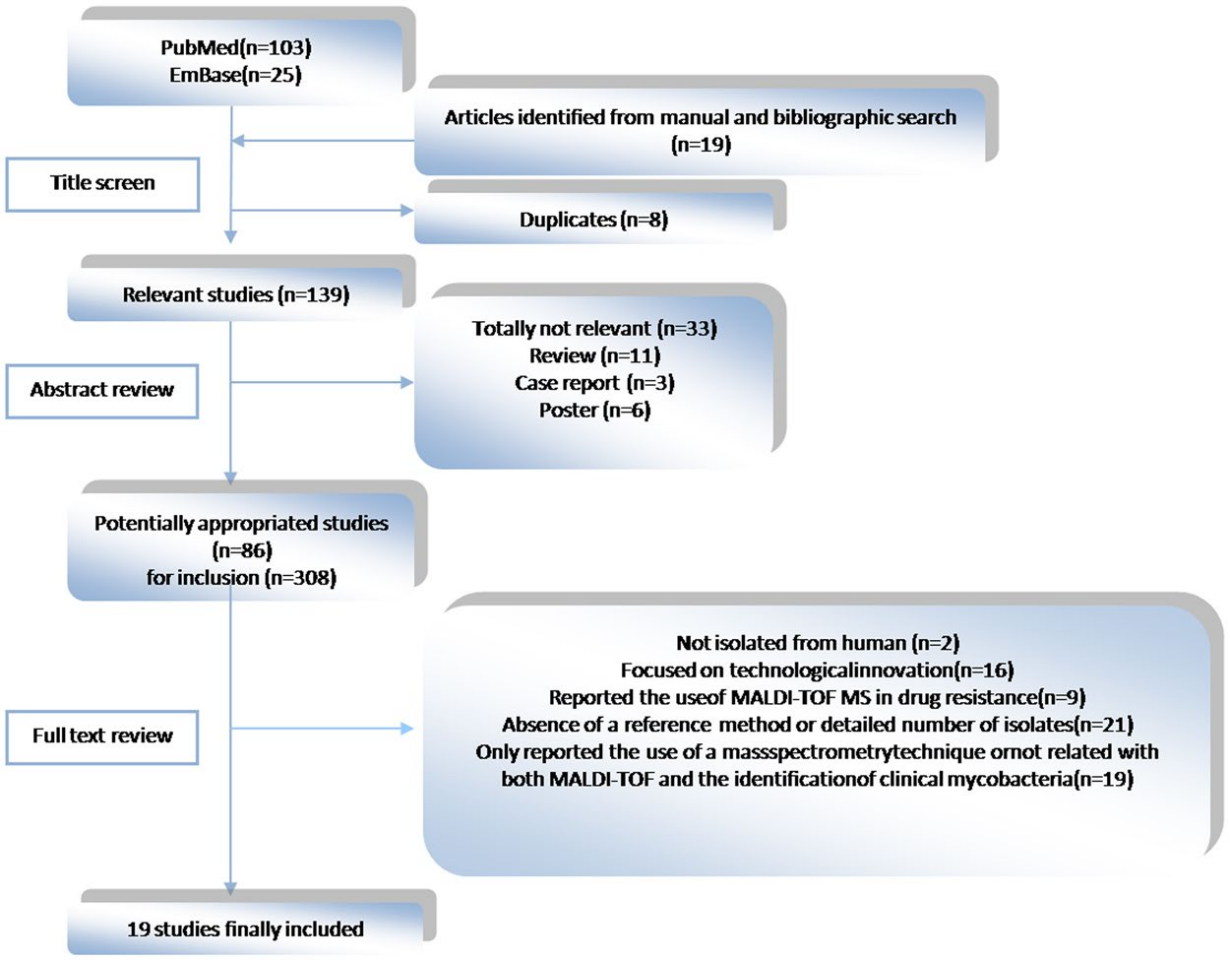
Flow diagram for systematic literature search.

Supplementary Table S1 showed the major characteristics of the enrolled studies. Among the 19 studies, two were prospective^15,16^. Six studies^17–22^ included reference strains, while other studies used only clinical isolates. Seven reports expanded an existing database by establishing reference spectra for clinical isolates^15–17,20,23–25^, while others used the databases from instrument suppliers. Only two articles clearly stated that a blinded method was used for their investigation^18,20^. The others did not specify use of a blinded method. Ten articles focused on identification of mycobacteria from solid cultures, while four incorporated both liquid and solid media cultures in the routine clinical microbiology setting^17,19,26,27^. Four studies evaluated the performance both of the Bruker Biotyper and Vitek MS MALDI-TOF MS systems for the identification of Mycobacterium^24,28–30^, while the others investigated only one or the other^31–33^.

### Overall meta-analysis

**Figure 2:**
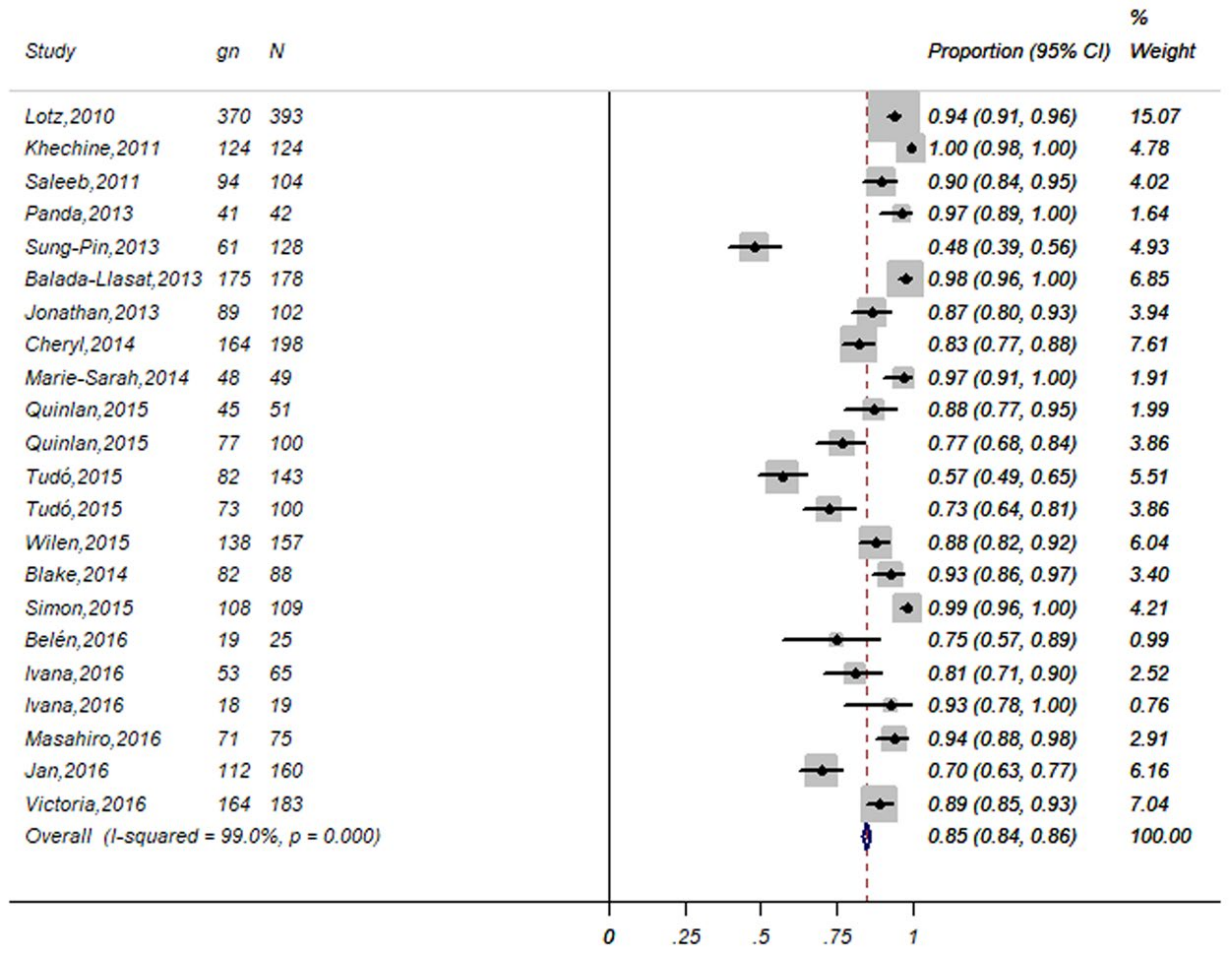
Forest plot for the meta-analysis of the gross identification ratio at the genus level. CI, confidence interval; W, weight; gn, number of correct identifications; N, total number of identifications.

**Figure 3:**
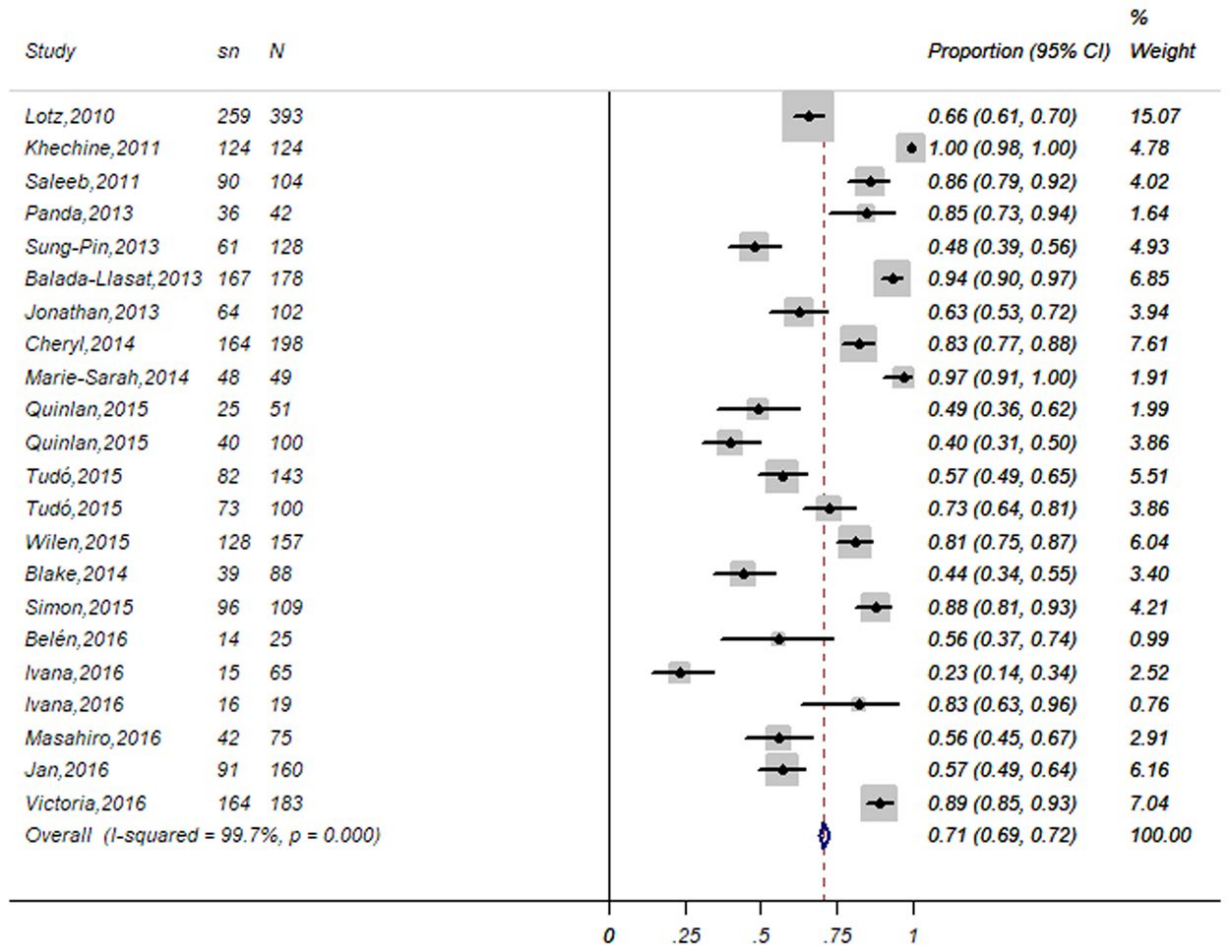
Forest plot for the meta-analysis of the gross identification ratio at the species level. CI, confidence interval; W, weight; sn, number of correct identifications; N, total number of identifications.

In the 19 enrolled studies, a total of 2,593 mycobacteria isolates were assessed. The overall statistical results of the meta-analysis at the genus and species level identification were summarized by forest plots of random-effects model (Figs 2 and 3). The gross correct identification ratios of MALDI-TOFMS for clinical mycobacteria ranged from 48% to 100% at the genus level and from 23% to 100% at the species level. Significant heterogeneity was found both at the genus level (P < 0.001; I^2^ = 99%) and the species level (P < 0.001; I^2^ = 99.7%). Of these, 2034 (85%; 95% CI of 84% to 0.86%) were correctly identified to the genus level while 1,841 (71%; 95% CI of 69% to 73%) were correctly identified to the species level by MALDI-TOF MS with random-effects model.

The pooled identification results of MALDI-TOF MS by random-effects for the majority of *Mycobacterium* species was shown in Table 1. *M. tuberculosis*, the most important cause of tuberculosis, showed a high identification proportion at 92% with a 95% CI of 87% to 96%. As another member of MTC, *M.bovis* had a moderate identification proportion at 68% with a 95% CI of 27% to 100%. In NTM family, identification accuracy of *M. haemophilum* was the highest at 93% with a 95% CI of 87% to 100%, followed by *M. marinum*, *M. fortuitum*, *M. peregrinum*, *M. xenopi*, *M. immunogenum*, *M. g*ord*onae*, *M. smegmatis, M. abscessus*, *M. mucogenicum*, *M. kansasii* and *M. avium*with an identification proportion *abov*e 80%. Identification accuracy of *M. chelonae* and *M. lentiflavum and M. simiae* were similar with an overall correct identification ratio at 70%. The lowest performance of MALDI-TOF MS was in *M. malmoense* and *M. phlei*, at ≤50%. Approximately 60% of *M. intracellulare*, *M. maesiliense*, *M. neoaurum*, *M. parascrofulaceum*, *M. scrofulaceum* and *M. szulgai* were correctly identified to the species level.

**Table 1:** identification accuracy rate of common species.

	Proportion	95%CI	Weight%
*M. tuberculosis*	0.92	0.87–0.96	5.75
*M. bovis*	0.68	0.27–1.00	1.15
*M. abscessus*	0.82	0.75–0.88	7.64
*M. avium*	0.80	0.69–0.91	6.41
*M. chelonae*	0.78	0.69–0.86	6.61
*M. fortuitum*	0.88	0.83–0.93	7.22
*M. gordonae*	0.84	0.78–0.90	6.48
*M. haemophilum*	0.93	0.87–1.00	2.93
*M. intracellulare*	0.55	0.34–0.76	4.39
*M. immunogenum*	0.86	0.75–0.98	2.64
*M. kansasii*	0.81	0.75–0.88	6.52
*M. lentiflavum*	0.74	0.52–0.95	2.37
*M. marinum*	0.92	0.86–0.98	4.64
*M. malmoense*	0.42	0.13–0.71	1.59
*M. maesiliense*	0.54	0.09–0.99	1.50
*M. mucogenicum*	0.82	0.73–0.91	4.13
*M. neoaurum*	0.66	0.43–0.89	2.34
*M. parascrofulaceum*	0.68	0.00–0.15	1.87
*M. peregrinum*	0.88	0.80–0.97	2.03
*M. phlei*	0.40	0.13–0.68	2.03
*M. scrofulaceum*	0.69	0.51–0.88	3.16
*M. smegmatis*	0.84	0.75–0.94	2.91
*M. simiae*	0.74	0.61–0.86	4.13
*M. szulgai*	0.69	0.53–0.86	3.77
*M. xenopi*	0.87	0.80–0.94	5.78
Total	0.74	0.71–0.79	100

### Subgroup meta-analyses

The heterogeneity and random-effects pooled ratios of subgroup analyses performed at the species level according to strain source (clinical isolates only or reference strains also), system database (commercial database only or self-established database also), system (Bruker MALDI Biotyper and the bioMérieuxVitek MS), culture media(liquid or solid), growth rate (fast or slow) and category of strain (MTC or NTM) are shown in Table 2. The correct identification ratios of MTC at the species level was 90% (95% CI of 86% to 94%), obviously higher than NTM groups at 74% with a 95% CI of 71% to 79%. The correct identification of bioMérieuxVitek MS slightly exceeded Bruker MALDI Biotyper (75% vs 72%), especially in the identification of *M. abscessus*, *M. avium*, *M. chelonae*, *M. immunogenum*, *M. intracellulare*, *M. mucogenicum*, *M. scrofulaceum*, *M. simiae* and *M. szulgai*, as shown in Table 3. No significant difference was observed between rapid and slow growing isolates, similar to the correct identification performances of overall meta-analysis. The correct identification performances of the sub-analyses on isolates on solid culture media, with reference strains and self-established database added outcomes were superior to the gross ratio in our meta-analysis and their respective compared group. However, the heterogeneity was not obviously decreased in subgroup meta-analyses.

**Table 2:** The heterogeneity and pooled correct identification ratios in subgroup analysis.

Subanalysis	No. of isolates	Within-group heterogeneity		Correct identification ratio (95%CI)
		*P*	*I* ^2^	
**System database**				
Commercial database only	1299	<0.001	98.0%	0.70(0.61–0.78)
Commercial database plus self-established database	1212	<0.001	96.8%	0.82(0.72–0.92)
**Source of strain**				
Clinical isolates only	1746	<0.001	96.8%	0.67(0.557–0.78)
Clinical isolates plus reference strains	765	<0.001	98.5%	0.77(0.63–0.91)
**Culture media**				
liquid	458	<0.001	85.4%	0.57(0.45–0.68)
solid	1734	<0.001	95.3%	0.70(0.59–0.74)
**Growth rate**				
fast	1119	<0.001	91.9%	0.75(0.71–0.80)
slow	1392	<0.001	93.4%	0.76(0.73–0.79)
**Category of strain**				
MTC	454	<0.001	91.9%	0.90(0.86–0.94)
NTM	2057	<0.001	97.9%	0.74(0.71–0.79)
**System**				
Bruker MALDI Biotyper	1801	<0.001	91.8%	0.72(0.62–0.81)
bioMérieux Vitek MS	786	<0.001	95.4%	0.75(0.68–0.82)

**Table 3:** comparison accuracy rate of two systems.

Species	System	
	Bruker MALDI Biotyper	bioMérieux Vitek MS
*M. abscessus*	0.98(0.95–1.00)	0.75(0.64–0.86)
*M. avium*	0.96(0.91–1.00)	0.74(0.63–0.85)
*M. chelonae*	0.97(0.93–1.00)	0.68(0.52–0.84)
*M. fortuitum*	0.85(0.72–0.99)	0.94(0.91–0.98)
*M. gordonae*	0.79(0.66–0.92)	0.77(0.66–0.87)
*M. haemophilum*	0.95(0.85–1.00)	0.93(0.81–1.00)
*M. immunogenum*	0.92(0.81–1.00)	0.81(0.59–1.00)
*M. intracellulare*	0.77(0.59–0.95)	0.63(0.43–0.84)
*M. kansasii*	0.59(0.14–1.00)	0.71(0.58–0.84)
*M. marinum*	0.88(0.73–1.00)	0.96(0.91–1.00)
*M. mucogenicum*	0.95(0.88–1.00)	0.65(0.47–0.83)
*M. scrofulaceum*	0.75(0.56–0.93)	0.32(0.00–0.67)
*M. simiae*	0.75(0.61–0.99)	0.61(0.28–0.94)
*M. szulgai*	0.75(0.61–0.99)	0.55(0.30–0.80)
*M. terrae*	0.33(0.00–0.65)	0.46(0.15–0.61)
*M. xenopi*	0.82(0.59–1.00)	0.77(0.65–0.88)

### Common misidentification pattern in these studies

Some species were not differentiated from each other very well by existing MALDI-TOF MS systems and commercial or laboratory-established databases including *M. abscessus* and *M. massiliense*, *M. fortuitum* and *M. septicum*, *M. mucogenicum* and *M. phocaicum*, *M. parascrofulaceum* and *M. scrofulaceum* (Table 4).

**Table 4:** Common misidentification pattern in these studies.

Sequence identification	MALDI-TOF MS identification	System	Reference
*M. abscessus*	*M. massiliense*	Bruker MALDI Biotyper	^20,23^
	*M. tuberculosis*	Bruker MALDI Biotyper	^16^
*M aubagnense*	*M phocaicum*	Bruker MALDI Biotyper	^27^
*M. avium*	*M. intracellulare*	Bruker MALDI Biotyper	^26^
*M. chelonae*	*M. immunogenum/M. abscessus*	Bruker MALDI Biotyper	^29^
*M. chimaera*	*M. intracellulare*	Bruker MALDI Biotyper	^23^
*M. europaeum*	*M. scrofulaceum*	bioMérieux Vitek MS	^24^
*M. fortuitum*	*M. septicum*	Bruker MALDI Biotyper	^19,26^
	*M. abscessus/M. chelonae*	Bruker MALDI Biotyper	^19^
*M. franklinii*	*M. chelonae*	bioMérieux Vitek MS	^16^
*M. intracellulare*	*M. avium/M. abscessus*	Bruker MALDI Biotyper	^16^
	*M.chimaera*	Bruker MALDI Biotyper	^26^
*M. immunogenum*	*M abscessus*	Bruker MALDI Biotyper	^27^
*M. llatzerense*	*M phocaicum*	Bruker MALDI Biotyper	^27^
*M. malmoense*	*M. tuberculosis*	Bruker MALDI Biotyper	^16^
*M mucogenicum*	*M phocaicum*	Bruker MALDI Biotyper	^19,23,27^
*M. nebraskensec*	*M. avium*	Bruker MALDI Biotyper	^28^
*M. novocastrense*	*M. austro/M. africanum*	Bruker MALDI Biotyper	^28^
*M. paraffinicumc*	*M. bovis/M. avium/M. intracellulare*	Bruker MALDI Biotyper	^28^
*M. parascrofulaceum*	*M. scrofulaceum*	Bruker MALDI Biotyper/ bioMérieux Vitek MS	^29^
	*M. avium*	Bruker MALDI Biotyper	^28^
*M phocaicum*	*M mucogenicum*	Bruker MALDI Biotyper	^27^
*M porcinum*	*M. peregrinum/M conceptionense/M fortuitum*	Bruker MALDI Biotyper	^27^
*M pseudoshottsii*	*M. marinum*	Bruker MALDI Biotyper	^27^
*M. terrae*	*M. arupense*	bioMérieux Vitek MS	^24^
*M. tusciae*	*M. vaccae*	Bruker MALDI Biotyper	^28^

### Assessment of publication bias and influence analysis

Little publication bias was detected at the species level by Begg rank correlation (with continuity correction) and Egger’s linear regression test of funnel plot asymmetry in this meta-analysis (z = −0.90 and P = 0.367 for Begg; t = −0.70 and P = 0.492 for Egger’s, see Supplementary Fig. S1).

Influence analysis showed that no individual study had any obvious influence on the combined gross ratio at the species level (see Supplementary Fig. S2).

## Discussion

As an recent technology for the clinical identification of microorganisms, MALDI-TOF MS has many advantages over other current methods^34^. In this study, we performed a systematic review and meta-analysis of the current literature assessing diagnostic performance of MALDI-TOF MS in clinical applications. According to the inclusion and exclusion criteria, a total of 19 related articles were used in this review. The pooled result agreed with the reference method was 85% identification of the isolates to genus level, and 71% to the species level respectively, which still cannot meet with the need of clinical microbiology diagnostics so far. In these articles, we mainly focused on 25 mycobacterial species that are frequently isolated in clinical microbiology laboratories. The pooled identification ratio of these species was 74% with a 95% CI of 71% to 79%. Many reports have demonstrated the application of MALDI-TOF mass spectrometry in clinical diagnostic microbiology, including anaerobic bacteria, *enterobacteriaceae*, gram-positive aerobic bacteria, non-enterobacteriaceae gram-negative bacilli, yeasts and so on, showing correct identification ratio at species level above 77%^35–39^. Our previous study showed that MALDI-TOF MS correctly identified 96% of the *streptococci* and 99% of the *Streptococcus pneumonia* to species level^9^, much higher than the performance for mycobacteria in this study. In contrast to other bacteria, the cell walls of mycobacterial species contain variable amounts of mycolic acids, resulting in awaxy, hydrophobic structure^40^. Some of the studies included in this evaluation analyzed whole cells, while others followed the cell extracts procedure. It should be noted that different cell extract procedures would impact the MS spectra generated, leading to inconsistencies within databases and poorer identification performance. In addition to cell extraction procedures, identification ratios of MALDI-TOF MS can be affected by other variables (e.g., the grow rate of strain, the proportion of clinical and reference species, or the culture media) that were revealed by heterogeneity in subgroup analysis.

According to our results, no notable differences in the overall identification rates between rapid slowly-growing mycobcteria. A high number of replicates increase the probability of correct identification, especially for slowly-growing mycobacteria. In some cases, five replicates were required to obtain one good spectral acquisition^17^. The identification accuracy of MTC was higher than NTM partially because most studies were more interested in MTC. As frequently detected pathogenic *Mycobacterium* species, MTC have a facility for protein profile acquisition in existing databases, leading to more accurate identification than for NTM. Since NTM are attracting attention due to increase in the isolation frequency, especially in the countries with declining tuberculosis incidence^41^, the identification ratio of NTM may increase as databases expand to include more isolates.

In our study, different isolation media commonly used in laboratory for the recovery of mycobacteria gave different results. Although it is more convenient to use isolated colonies from solid media for MS analysis^42^, mycobacterial identification from liquid cultures can accelerate pathogen identification prior to growth on solid media^43,44^. There remnants of nutrient substances from liquid medium do not interfere substantially with the pattern of the mycobacterial spectra or impair the identification rate of VITKS MS when the modified protocol for processing liquid cultures, including a second ethanol washing step is used. Nevertheless, the percentage of isolates identified with a low confidence level (75–85%) was higher from liquid medium compared to solid medium, even when using a second ethanol washing step^22^. However, Aure´lieLotz *et al*. determined that identification results from growth in liquid medium were not as good as those obtained from solid medium either due to the low number of bacteria or to potential interference of the supplements such as PANTA and OADC included in the complex medium^17^.

In the meantime, we noticed differences in identification capabilities of the two commercial MALDI-TOFMS systems (the Bruker MALDI Biotyper and the bioMérieuxVitek MS). There are, in all, four reports comparing the two systems. Mather *et al*. used two simplified protein extraction protocols at the University Of Washington (UW) and by bioMérieux and both mass spectrometry platforms. Their results demonstrated that the identification performance of bioMérieuxVitek MS was better than Bruker MALDI Biotyper no matter which protocol was used by no naugmented database^24^. The report of Lévesque *et al*. indicated that the bioMérieux VITEK MS correctly identified more mycobacteria to species level than Bruker Biotyper^30^. However, Wilen and colleagues compared two MALDI-TOF MS instrumentation platforms and three databases: Bruker Biotyper Real Time Classification3.1 (Biotyper), Vitek MS Plus Saramis Premium (Saramis), and Vitek MS v3.0 (28). The levels of accuracy were not significantly different across the three platforms. However, the Vitek MS v3.0 database could offer modest advantages over the Biotyper and Saramis, especially by reducing the necessity of repeat identification attempts^29^.

It comes as no surprise that the identification accuracy of the clinical isolates plus reference strains was higher than that of the clinical isolates alone. This is because reference strains were more likely to have matching spectra in existing databases because of their inherent stable spectral profiles. The misidentification issues in Table 4 could be due to inherent unsatisfactory spectra from these species for highly similar spectral profiles from closely related species or subspecies within a complex, or insufficient numbers of spectra for uncommon species in reference libraries. Thus, it will be increasingly important to update these libraries include more reference spectra as well as the optimized extraction methods used to create the spectra.

Last but not least, there are still some limitations in our study. Firstly, Table 1 not showed all mycobacteria species because data of species with more than three reports were recalculate in this study for statistical reason. Moreover, some articles reported that MALDI-TOF MS identified isolates to “complex” level, such as “*M. fortuitum* complex”. We refer this situation as genus level; this may partially underestimate the accuracy of MALDI-TOF MS for identification of Mycobacteria to species level.

Different databases and system, differences in the preparation of sample spectra, and the composition of the species included in the study are probably responsible for observed differences in the overall identification rates in these studies. Despite our study demonstrated the use of MALDI-TOF MS as less reliable technique for the accurate identification of mycobacterial species, with the introduction of more spectra of representative organisms into the identification database and the development of a refined methodology, MALDI-TOFMS has become a promising tool for the identification of clinical pathogens to initiate early treatment and thus prevent drug resistance. Future studies to analyze the comprehensive capability of this technology for clinical microbiology diagnostics are warranted.

## Materials and Methods

### Search strategy

We queried PubMed (up to 1th March 2017) with the string “(maldi-ms [MeSH Terms] AND mycobacteria [MeSH Terms]) AND (identification [Title/Abstract] OR detection [Title/Abstract])” to identify relevant articles. We also searched Embase database with the words “maldi tof mass spectrometry,” “mycobacteria”, “mycobacterium,” “identification,” and “detection” with no language, publication status, and geographical distribution restriction. Two investigators (Yan Cao, Lei Wang) performed the literature search and data extraction independently. Disagreements were resolved by discussion and/or consultation with a third researcher (Bing Gu).

### Study selection criteria and data extraction

The inclusion and exclusion criteria were established by the investigators prior to the review of literature. The accuracy of MALDI-TOF MS for identification of clinical mycobacteria isolates confirmed by gold standard methods was considered eligible for the meta-analysis. Studies or data were excluded as follows: case reports/reviews/posters; studies applying MALDI-TOF MS to identify industrial/environmental isolates; studies on technological innovations; in drug resistance; lack of a reference method or detailed number of isolates. The numbers of isolates correctly identified and of total isolates at the genus and species levels were abstracted according to the category of strain, the MS system or database used, and the culture method used.

### Quality assessment

The quality of eligible studies was assessed by using the Quality Assessment of Diagnostic Accuracy Studies(QUADAS) guide lines^45^ to assess the quality of original studies: study design, system database, reference methods, category of strains, and blinded status (see Supplementary Table 1).

### Data synthesis and analysis

The identification ratio was calculated as the number of correctly identified isolates divided by the total number of isolates^46^. The double arcsine-transformed ratios were subsequently pooled in random-effects model when significant heterogeneity was present. Pooled transformed estimate formulas were back-transformed into the original ratios^47^ for better understanding. Subgroup analyses at the species level were performed according to: strain, culture media, source of strain, and system database.

I^2^ measure was used to estimate heterogeneity between studies. The rank correlation method of Begg and Egger’s regression were used to evaluate publication bias^48^. All analyses were performed with Stata Statistical Software Package, version 1 1.0 (Stata Corp LP, College Station USA).

## Electronic supplementary material

Supplementary Information
