# Supplementary material for: Accuracy of Matrix-Assisted Laser Desorption Ionization–Time of Flight Mass Spectrometry for Identification of Mycobacteria: a systematic review and meta-analysis
Source: Sci Rep. 2018 Mar 7;8:4131. doi: 10.1038/s41598-018-22642-w (PMC5841357; doi:10.1038/s41598-018-22642-w)
Supplement: Supplementary file 1 — Supplementary Information [file 41598_2018_22642_MOESM1_ESM.pdf]

Supplementary information

Accuracy of Matrix-Assisted Laser Desorption Ionization–Time of Flight Mass Spectrometry for Identification of Mycobacteria: a systematic review and meta-analysis

Yan Cao [1*], Lei Wang[2*], Ping Ma[3, 4], Fan Wenting[4], Bing Gu[#3, 4], Shaoqing Ju[#1]

1. Center of Laboratory Medicine, Affiliated Hospital of Nantong University, Nantong 226000, China.
2. Department of Histology and Embryology, Xuzhou Medical University, Xuzhou 221004, China.
3. Department of Laboratory Medicine, Affiliated Hospital of Xuzhou Medical University, Xuzhou 221002, China.
4. Medical Technology School, Xuzhou Medical University, Xuzhou 221004, China.

[*]: The first two authors contributed equally to this work.

[#]Corresponding author:

Bing Gu, Department of Laboratory Medicine, Affiliated Hospital of Xuzhou Medical University, Xuzhou 221002, China.E-mail: gb20031129@163.com, Phone: +86-0516-83262509.

Shaoqing Ju, Department of Laboratory Medicine, Affiliated Hospital of Nantong University, Nantong 226000, China. E-mail: jsq814@hotmail.com, Tel/Fax:+86-0513-85052610

Table S1. Characteristics of the 19 reports in this meta-analysis

Study	Study design	System	Self-established database	Reference strains	QUADAS quality
Lotz,2010	retrospective	Biotyper	Yes	Yes	11
Khechine,2011	prospective	Biotyper	Yes	No	12
Saleeb,2011	retrospective	Biotyper	Yes	No	11
Panda,2013	retrospective	Biotyper	No	Yes	12
Sung-Pin,2013	retrospective	Biotyper	No	No	11
Balada-Llasat,2013	retrospective	Biotyper	No	Yes	11
Jonathan,2013	retrospective	Biotyper/Vitek MS	No	No	12
Cheryl,2014	retrospective	Biotyper/Vitek MS	Yes	No	10.5
Marie-Sarah,2014	retrospective	Biotyper	Yes	Yes	11.5
Blake,2014	retrospective	Biotyper	No	No	11
Quinlan,2015	retrospective	Biotyper	No	No	11
Tudó,2015	prospective	Biotyper	Yes	No	12
Wilen,2015	retrospective	Biotyper/Vitek MS	No	No	11
Simon,2015	retrospective	Biotyper/Vitek MS	No	No	11
Belén,2016	retrospective	Biotyper	No	Yes	11
Ivana,2016	retrospective	Biotyper	No	No	11
Masahiro,2016	retrospective	Biotyper	No	No	12
Jan,2016	retrospective	Vitek MS	No	Yes	12
Victoria,2016	retrospective	Vitek MS	Yes	No	12

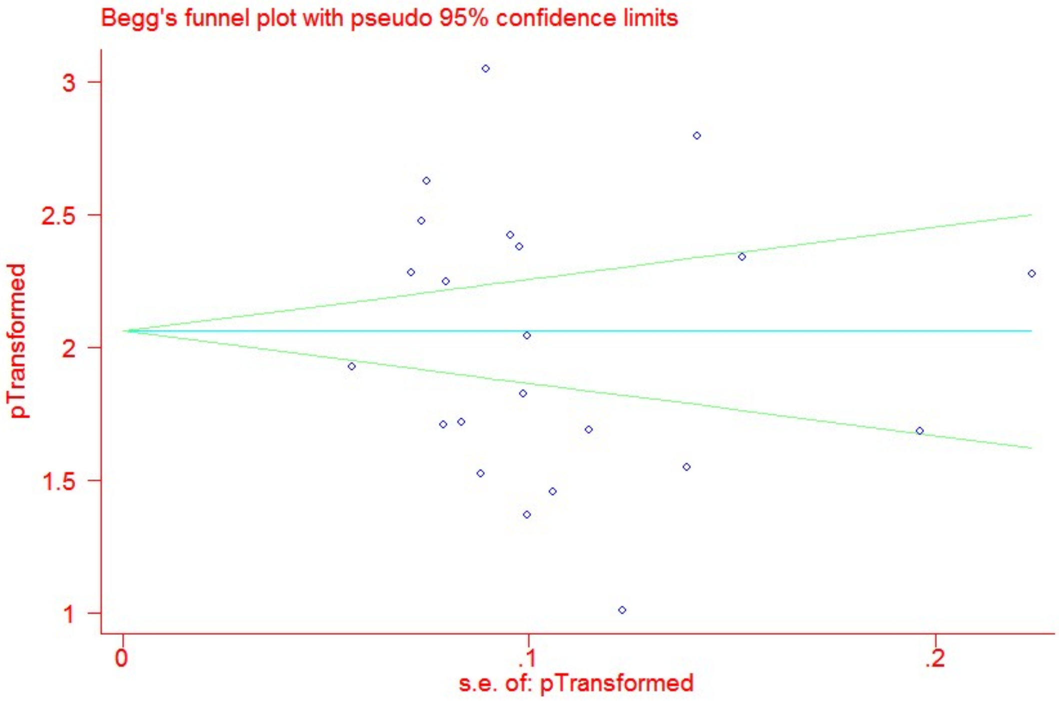

Supplementary Figure 1. Funnel plot asymmetry at the species level

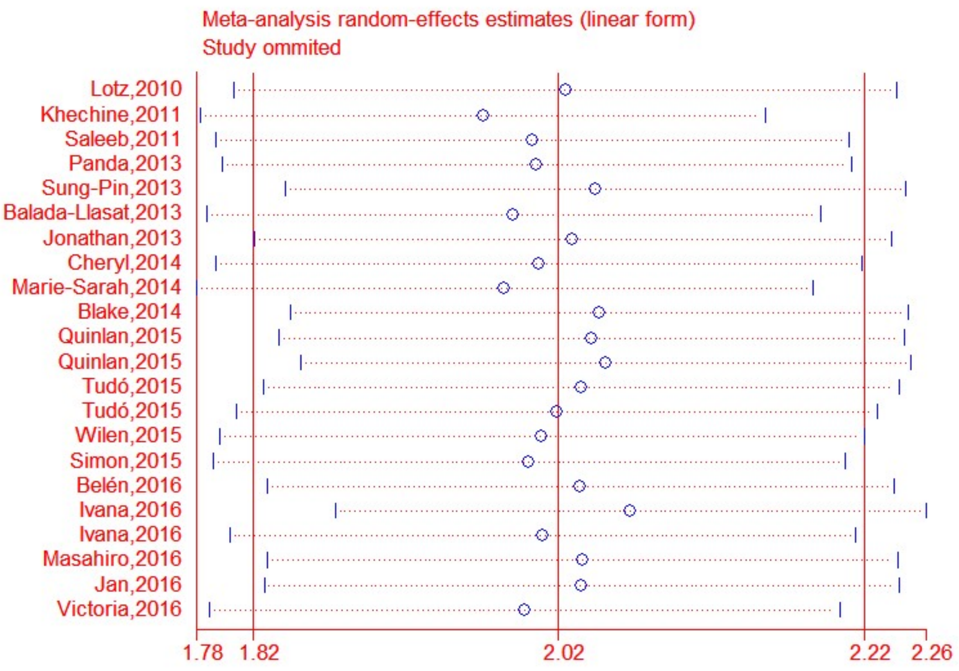

Supplementary Figure 2. Influence analysis with a random-effects model for the enrolled articles at the species level.
